# The Relationship between Serum CXCL8 and ET-1 Expression Levels and Sepsis Complicated with Heart Failure

**DOI:** 10.1155/2022/8570486

**Published:** 2022-08-27

**Authors:** Jianlong Zhu, Changjun Song, Tingting Cai, Lulu Yi, Wei Zhang, Jing Zhong, Meirong Shen

**Affiliations:** ^1^Department of Critical Care Medicine, Ganzhou People's Hospital, Ganzhou 341000, China; ^2^Department of Emergency, Ganzhou People's Hospital, Ganzhou 341000, China; ^3^Department of Infectious Diseases, Ganzhou People's Hospital, Ganzhou 341000, China; ^4^Department of Rheumatology, Ganzhou People's Hospital, Ganzhou 341000, China; ^5^Department of Critical Care Medicine, Quannan People's Hospital, Ganzhou 341000, China

## Abstract

**Objective:**

The objective is to investigate the relationship between sepsis complicated with heart failure and the expression levels of CXC chemokine ligand 8 (CXCL8) and endothelin-1 (ET-1).

**Methods:**

A total of 128 sepsis patients accepted by the Ganzhou People's Hospital from March 2019 to December 2021 were collected as observation objects, and they were separated into a simple sepsis group (86 cases) and a complicated heart failure group (42 cases) according to whether they were accompanied by heart failure or not. General data such as Sequential Organ Failure Assessment (SOFA) score and Acute Physiology and Chronic Health Evaluation II (APACHE II) were collected; the expression levels of serum CXCL8 and ET-1 were detected by enzyme-linked immunosorbent assay (ELISA); the cardiac function parameters such as left ventricular ejection fraction (LVEF), stroke volume (SV), cardiac output (CO), and cardiac index (CI) were measured by color Doppler ultrasound; the correlation between serum CXCL8 and ET-1 expression levels with clinical data and cardiac function parameters in patients with sepsis complicated with heart failure was analyzed by the Pearson correlation; and the influencing factors of sepsis complicated with heart failure were analyzed by the logistic regression analysis.

**Results:**

The serum CXCL8 and ET-1 expression levels, SOFA score, and APACHE II score in the complicated heart failure group were higher than those in the simple sepsis group (*P* < 0.05), and LVEF, SV, CO, and CI in the complicated heart failure group were lower than those in the simple sepsis group (*P* < 0.05). Serum CXCL8 was positively correlated with ET-1 in patients with sepsis complicated with heart failure (*r* = 0.531, *P* < 0.05), and the two were positively correlated with SOFA score and APACHE II score (*P* < 0.05) and were negatively correlated with LVEF, SV, CO, and CI (*P* < 0.05). CXCL8 and ET-1 were independent risk factors for sepsis complicated with heart failure (*P* < 0.05).

**Conclusion:**

The expression levels of serum CXCL8 and ET-1 in sepsis patients with heart failure are significantly increased, and both are risk factors for heart failure in sepsis patients.

## 1. Introduction

Sepsis can be broadly defined as a systemic response to microbial infection. It is a life-threatening organ dysfunction caused by the maladjustment of the host response to infection [1, 2]. Myocardial dysfunction is one of the common complications of patients with sepsis. Patients usually have acute onset and rapid progress; the pathogenesis of myocardial disease caused by sepsis involves mitochondrial dysfunction, dysregulation of inflammatory mediators, oxidative stress, endothelial dysfunction, etc. [3]. Looking for early markers related to sepsis complicated with heart failure may help to improve the therapeutic effect. C-X-C motif chemokine ligand 8 (CXCL8), also known as interleukin-8 (IL-8), is one of the most important proinflammatory factors and plays a vital role in many inflammatory diseases including ulcerative colitis [4, 5]. Zhao et al. [6] showed that the −251 A/T polymorphism of IL-8 gene may affect the susceptibility of individuals to sepsis. Endothelin-1 (ET-1) is the most common form of ET, originally called pure vasoconstrictor, which plays an important role in the pathogenesis of different cardiovascular diseases. Its role in pathophysiology of ischemia/reperfusion injury has been fully confirmed [7]. However, there are few reports about whether CXCL8 and ET-1 are related to sepsis complicated with heart failure. In view of this, this study aims to provide a reference for disease prevention by detecting serum CXCL8, ET-1, and cardiac function parameters in sepsis patients complicated with heart failure, and analyzing the correlation between these indexes.

## 2. General Information and Methods

### 2.1. General Information

A total of 128 patients with sepsis admitted to the Ganzhou People's Hospital from March 2019 to December 2021 were selected as observation objects. According to whether they were accompanied by heart failure or not, they were divided into a simple sepsis group (86 cases) and a complicated heart failure group (42 cases). Among them, the patients in the simple sepsis group were 31–84 years old and the patients in the complicated heart failure group were 32–83 years old. The medical records of all patients were consulted and collected, including age, sex, body mass index (BMI), body temperature, ICU length of stay, invasive mechanical ventilation, renal replacement therapy, comorbidities (hypertension, diabetes, and chronic obstructive pulmonary disease), Sequential Organ Failure Assessment (SOFA) score, and Acute Physiology and Chronic Health Evaluation II (APACHE II) score.

**Figure 1:**
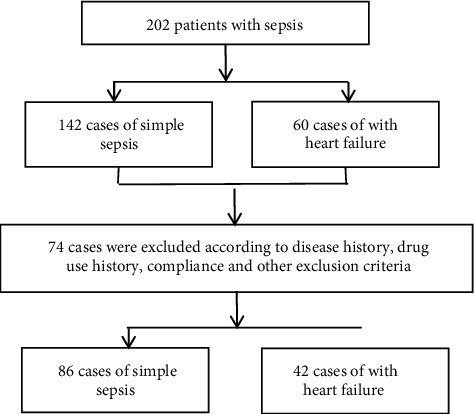
Case exclusion flow chart.

Inclusion criteria were as follows: ① sepsis patients who meet the standards in the international management guidelines for sepsis/septic shock [8] when admitted, and patients complicated with heart failure who meet the relevant standards formulated by the Clinical Data Standards Working Group of the American Heart Association/American Heart Association [9]; ② patients with complete clinical data; and ③ patients or family members who have given informed consent to the study, which has been approved by the hospital clinical ethics committee, and signed a paper consent form. Exclusion criteria were as follows: ① patients with a previous history of heart disease; ② patients with infectious diseases, autoimmune diseases, and blood system diseases; ③ patients with mental illness or poor compliance; and ④ patients with a history of surgery or other medication in the past month. The case exclusion flow chart is shown in Figure 1.

Diagnosis of heart failure with reduced ejection fraction: there is the presence of dyspnea or exertional, nocturnal, paroxysmal dyspnea, and other manifestations; patients are often accompanied by lower extremity edema, fatigue, and other symptoms; signs include pulmonary rales, lower extremity edema, and typical symptoms and signs of heart failure, such as jugular vein filling and positive hepatic jugular vein reflux sign; and echocardiography shows an ejection fraction <40%, and increased brain natriuretic peptide or *N*-terminal brain natriuretic peptide precursor. Diagnosis of heart failure in the middle range of ejection fraction: there are typical symptoms or signs of heart failure; echocardiography shows an ejection fraction of 40%–50%, accompanied by the performance of a decreased diastolic function or changes in ventricular wall thickening; and elevation of brain natriuretic peptide or *N*-terminal brain natriuretic peptide precursor. Diagnosis of heart failure with preserved ejection fraction: there is the presence of typical symptoms or signs of heart failure; echocardiography shows ejection fraction >50%, accompanied by a decreased diastolic function or evidence of ventricular wall thickening; brain natriuretic peptide; or *N* elevation of terminal brain natriuretic peptide precursor.

### 2.2. Research Methods

#### 2.2.1. Detection of Serum CXCL8 and ET-1 Expression Levels

5 mL peripheral venous blood samples were from patients on an empty stomach in the morning and centrifuged at 3000 r/min for 15 minutes. The upper serum was separated and collected, and stored in the refrigerator at −80°C. Enzyme-linked immunosorbent assay (ELISA) was used to detect the expression levels of CXCL8 (CXCL8 kit purchased from Beijing Solarbio Technology Co., Ltd.) and ET-1 (ET-1 kit purchased from Beijing Bowers Biotechnology Co., Ltd.) in patients' serum, and the detection process was carried out according to the kit and instrument instructions.

#### 2.2.2. Detection of Cardiac Function Parameters

The Philips IU22 color Doppler ultrasound instrument (Dutch Philips Company) was used to detect cardiac function parameters, including left ventricular ejection fraction (LVEF), stroke volume (SV), cardiac output (CO), and cardiac index (CI).

### 2.3. Statistical Analysis

The SPSS 22.0 software package was selected to input and analyze all data, in which the measurement data were expressed by ($\bar{x}\pm s$). The independent-sample *t* test was performed. The counting data were expressed by *n* (%), and the chi-squared test was performed; Pearson's correlation was used to analyze the correlation between the expression levels of serum CXCL8 and ET-1 and clinical data and cardiac function parameters in sepsis patients complicated with heart failure. A logistic regression analysis of influencing factors of sepsis complicated with heart failure was carried out. *P* < 0.05 indicated a statistically significant difference.

## 3. Results

### 3.1. Comparison of Clinical Data between the Simple Sepsis Group and the Complicated Heart Failure Group

There was no significant difference in age, sex, BMI, body temperature, ICU length of stay, invasive mechanical ventilation, renal replacement therapy, hypertension, diabetes, and chronic obstructive pulmonary disease between the complicated heart failure group and the simple sepsis group (*P* > 0.05). The SOFA score and APACHE II score of patients complicated with heart failure were higher than those of patients with sepsis alone (*P* < 0.05; see Table 1).

### 3.2. Comparison of Cardiac Function Parameters between the Simple Sepsis Group and the Complicated Heart Failure Group

LVEF, SV, CO, and CI in patients complicated with heart failure were lower than those in patients with sepsis alone (*P* < 0.05; see Table 2).

### 3.3. Comparison of Serum CXCL8 and ET-1 Expression Levels between the Simple Sepsis Group and the Complicated Heart Failure Group

The expression levels of serum CXCL8 and ET-1 in patients complicated with heart failure were higher than those in patients with sepsis alone (*P* < 0.05; see Table 3).

### 3.4. Correlation between the Expression Levels of Serum CXCL8 and ET-1 in Sepsis Patients Complicated with Heart Failure and Clinical Data and Cardiac Function Parameters

**Figure 2:**
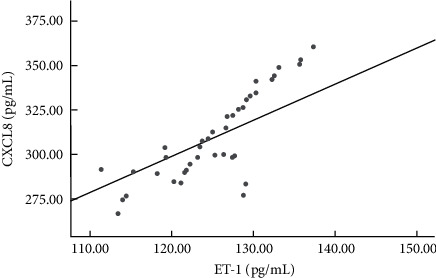
Correlation between serum CXCL8 and ET-1 in sepsis patients complicated with heart failure.

According to Pearson's correlation analysis, serum CXCL8 of sepsis patients complicated with heart failure was positively correlated with ET-1 (*r* = 0.531, *P* < 0.05). Both of them were positively correlated with SOFA score and APACHE II score (*P* < 0.05), and negatively correlated with LVEF, SV, CO, and CI (*P* < 0.05; see Figure 2 and Table 4).

### 3.5. Logistic Regression Analysis of Influencing Factors of Sepsis Complicated with Heart Failure

The multivariate logistic regression analysis was carried out with whether the sepsis patient is complicated with heart failure as a dependent variable and with CXCL8, ET-1, LVEF, SV, CO, CI, SOFA score, and APACHE II score as independent variables. The results showed that CXCL8 and ET-1 were independent risk factors for sepsis complicated with heart failure (*P* < 0.05; see Table 5).

## 4. Discussion

Sepsis and septic shock are medical emergencies. Sepsis 3.0 guidelines suggest that the disease should be treated and resuscitated immediately, including initial resuscitation within the first 3 hours and further hemodynamic assessment by echocardiography [10]. Myocardial insufficiency caused by sepsis is characterized by decreased left ventricular diastolic function and LVEF [11]. Sepsis complicated with heart failure is the main risk factor for sepsis patients' death. Once heart failure occurs, the mortality rate can obviously increase [12]. Therefore, early identification of sepsis complicated with heart failure in sepsis is vitally important. However, at present, the clinical obstacle is that there are no clear diagnostic criteria for sepsis complicated with heart failure. Ultrasonography is the most important examination method of cardiac dysfunction in sepsis patients, which is widely used. LVEF, SV, CO, and CI are commonly used cardiac function parameters, which can be used to evaluate patients' cardiac function. However, the limitations of each index have been verified by many literatures [13, 14].

CXCL8 is one of the earliest and most deeply studied chemokines. It is a typical CXC chemokine and can be released by many cell types, including neutrophils, monocytes, macrophages, fibroblasts, endothelial cells, and intestinal epithelial cells [15]. CXCL8 can hardly be detected in unstimulated cells or tissues. Yet if it is activated by various cytokines, even CXCL8 itself, its expression level will increase significantly [16]. Chen et al. [14] showed that the level of plasma IL-8 in sepsis patients with myocardial dysfunction was significantly higher than that in nonmyocardial dysfunction patients. The results showed that the expression level of CXCL8 in serum of patients with sepsis and heart failure was higher than that of patients with sepsis alone, which was consistent with those of previous studies. It is suggested that the increase of serum CXCL8 expression level is significantly related to the heart failure of sepsis patients. It is speculated that the pathophysiological cascade reaction begins with the response of some sepsis patients' immune systems to invading pathogens. Then, the innate immune response is activated, thus promoting the release of inflammatory mediators and signal molecules, and activating the positive feedback and negative feedback in the immune system. The increase in inflammatory factor CXCL8 leads to the damage of myocardial cells, which further promotes the occurrence of complicated heart failure in sepsis patients.

Endocardial endothelial cells affect the contractility of myocardial cells through paracrine signaling substances such as ET-1, angiotensin II, and nitric oxide [17]. Heart failure, atrial fibrillation, and ischemia/reperfusion injury are typical lesions of endocardial endothelium. These kinds of patients may have systemic endothelial dysfunction, which combines the dysfunction of endocardium and vascular endothelium and leads to the increase of hemodynamic load of the left atrium and the increase of synthesis and release of ET-1 and angiotensin II [18, 19]. In this study, the expression level of serum ET-1 in sepsis patients complicated with heart failure was significantly higher. It is suggested that the increase of ET-1 expression level may be an important sign of heart failure in sepsis patients. Combined with the correlation results of this study that ET-1 expression level was positively correlated with CXCL8, it is speculated that the possible reasons for the increase of ET-1 expression level are that pathogen invasion caused the increase in inflammatory reaction in sepsis patients and that the level of inflammatory factor CXCL8 increased significantly. Due to individual differences, some sepsis patients had endocardial and vascular endothelial dysfunction, leading to an increase in hemodynamic load and ET-1 detection level. To further verify the relationship between CXCL8, ET-1, and sepsis complicated with heart failure, Pearson's correlation analysis was used to verify the correlation between CXCL8, ET-1, and heart function parameters, SOFA score and APACHE II score. The results showed that CXCL8 and ET-1 were significantly correlated with heart function parameters, SOFA score and APACHE II score. Besides, CXCL8 and ET-1 were risk factors for sepsis patients complicated with heart failure in the logistic regression analysis. It is further suggested that CXCL8 and ET-1 can not only reflect the severity of sepsis patients, but also be used as important biomarkers for sepsis patients complicated with heart failure, and their predictive value needs further verification.

To sum up, the expression levels of serum CXCL8 and ET-1 in sepsis patients complicated with heart failure were upregulated, and their expressions were closely related to cardiac function parameters. CXCL8 and ET-1 may affect sepsis patients complicated with heart failure through a synergistic effect. CXCL8 and ET-1 may become potential therapeutic targets for sepsis complicated with heart failure, which is of great significance to the formulation and adjustment of treatment plan for patients. In the future, the predictive value of serum CXCL8 and ET-1 in sepsis patients complicated with heart failure will be further evaluated, and compared with ultrasonography to explore their clinical application value. In addition, the limitation of this study is that this study is only a small sample study, the included cases are all selected from the same hospital, and no clinical verification has been carried out. Therefore, a multicenter, large-sample prospective study will be used for verification in the future.

## Figures and Tables

**Table 1:** Comparison of clinical data between the simple sepsis group and the complicated heart failure group [$\left(\bar{x}\pm s\right)$/*n* (%)].

Index	Simple sepsis group (*n* = 86)	Complicated heart failure group (*n* = 42)	*t/χ* ^2^	*P*
Age (year)	55.42 ± 8.76	56.83 ± 9.12	0.844	0.400
Male [*n* (%)]	50 (58.14)	25 (59.52)	0.022	0.881
BMI (kg/m^2^)	23.81 ± 2.67	24.16 ± 3.02	0.667	0.506
Temperature (°C)	36.85 ± 1.02	36.73 ± 1.01	0.627	0.532
ICU length of stay (d)	7.94 ± 3.26	8.12 ± 3.29	0.292	0.770
Invasive mechanical ventilation [*n* (%)]	41 (47.67)	21 (50.00)	0.061	0.805
Renal replacement therapy [*n* (%)]	21 (24.42)	12 (28.57)	0.254	0.614
Hypertension [*n* (%)]	24 (27.91)	14 (33.33)	0.398	0.528
Diabetes [*n* (%)]	12 (13.95)	7 (16.67)	0.164	0.685
Chronic obstructive pulmonary disease [*n* (%)]	27 (31.40)	16 (38.10)	0.568	0.451
SOFA score (point)	7.45 ± 2.33	11.64 ± 1.69	10.387	0.000
Apache II score (point)	18.72 ± 3.51	24.67 ± 2.52	9.812	0.000

**Table 2:** Comparison of cardiac function parameters between the simple sepsis group and the complicated heart failure group.

Group	Number of cases (*n*)	LVEF (%)	SV (mL)	CO (L/min)	CI (min/m^2^)
Simple sepsis group	86	58.74 ± 16.82	58.44 ± 16.31	4.82 ± 1.39	3.89 ± 1.20
Complicated heart failure group	42	33.54 ± 10.28	40.07 ± 13.21	3.61 ± 1.13	2.24 ± 0.75
*t*	—	8.920	6.349	4.903	8.158
*P*	—	0.000	0.000	0.000	0.000

**Table 3:** Comparison of serum CXCL8 and ET-1 expression levels between the simple sepsis group and the complicated heart failure group.

Group	Number of cases (*n*)	CXCL8	ET-1
Simple sepsis group	86	254.16 ± 24.17	65.42 ± 6.46
Complicated heart failure group	42	306.75 ± 22.81	125.34 ± 7.62
*t*	—	11.770	46.406
*P*	—	0.000	0.000

**Table 4:** Correlation between expression levels of serum CXCL8 and ET-1 in sepsis patients complicated with heart failure and clinical data and cardiac function parameters.

Index	CXCL8		ET-1	
	*r*	*P*	*r*	*P*
LVEF	−0.526	0.000	−0.485	0.000
SV	−0.492	0.000	−0.496	0.000
CO	−0.489	0.000	−0.504	0.000
CI	−0.507	0.000	−0.491	0.000
SOFA score	0.467	0.000	0.504	0.000
APACHE II score	0.482	0.000	0.508	0.000

**Table 5:** Influencing factors of sepsis complicated with heart failure by logistic regression analysis.

Influencing factor	*B*	*SE*	*Wald*	*P*	*OR*	*95*% *CI*
CXCL8	0.922	0.328	7.906	0.005	2.515	1.322∼4.783
ET-1	0.867	0.316	7.522	0.006	2.379	1.281∼4.420
LVEF	0.098	0.120	0.667	0.414	1.103	0.872∼1.395
SV	0.265	0.251	1.118	0.290	1.304	0.797∼2.133
CO	0.190	0.228	0.693	0.405	1.209	0.773∼1.890
CI	0.143	0.137	1.093	0.296	1.154	0.882∼1.509
SOFA score	0.116	0.114	1.035	0.309	1.123	0.898∼1.404
Apache II score	0.142	0.246	0.335	0.563	1.153	0.712∼1.867

## Data Availability

The data used to support the findings of this study are included within the article (see the link of the public database https://doi.org/10.6084/m9.figshare.19447454.v1 for details).

## Abbreviations

CXCL8: C-X-C motif chemokine ligand 8
IL-8: interleukin-8
ET-1: endothelin-1
BMI: body mass index
SOFA: sequential Organ Failure Assessment
LVEF: left ventricular ejection fraction
SV: stroke volume
CO: Cardiac output
CI: cardiac index
APACHE II: Acute Physiology and Chronic Health Evaluation II.

## Additional Points

Research involving human participants and/or animal: This retrospective study involving human participants was in accordance with the ethical standards of the institutional research committee and with the 1964 Helsinki Declaration and its later amendments or comparable ethical standards. The study was approved by our institution's ethics review board.
